# A Simple Method for Prosthodontic Rehabilitation of Edentulous Patient with Epidermolysis Bullosa: A Clinical Case Report

**DOI:** 10.5681/joddd.2011.015

**Published:** 2011-06-14

**Authors:** Farhang Mahboub, Katayoun Sadr, Fateme Heidary, Elham Hosseini

**Affiliations:** ^1^ Assistant Professor, Department of Prosthodontics, Faculty of Dentistry, Tabriz University of Medical Sciences, Tabriz, Iran; ^2^ Dental and Periodontal Research Center, Faculty of Dentistry, Tabriz University of Medical Sciences, Tabriz, Iran; ^3^ Post-graduate Student, Department of Prosthodontics, Faculty of Dentistry, Tabriz University of Medical Sciences, Tabriz, Iran; ^4^ Post-graduate Student, Department of Pediatric Dentistry, Faculty of Dentistry, Tabriz University of Medical Sciences, Tabriz, Iran

**Keywords:** Complete denture, epidermolysis bullosa, impression, microstomia, rehabilitation

## Abstract

An abnormally small oral orifice is defined as microstomia. Microstomia may result from epidermolysis bullosa (EB), which consists of a group of disorders characterized by the presence of mechanical fragility of the skin with recurrent de-velopment of blisters and vesicles, resulting from minor mechanical friction or trauma. Since such patients have a small oral aperture, it may be impossible to take impression and fabricate dentures using conventional methods. In this article, a simple method for taking preliminary impressions from upper and lower edentulous ridges in one patient with limited mouth opening and then preparing the complete denture with custom denture teeth in a single unit was described.

## Introduction

An abnormally small oral orifice is referred to as microstomia.^1^ Microstomia may result from surgical treatment of orofacial neoplasms, cleft lips, maxillofacial traumas, burns, radiotherapy, scleroderma or genetic disorders such as epidermolysis bullosa and connective tissue diseases such as systemic sclerosis.^2-3^ Fragility and skin blistering are the hallmark features of the hereditary disorders classified as epidermolysis bullosa (EB). Although the specific pathogenesis of these disorders remains unknown, bulla formation has been associated with numerous basic defects including structural or biochemical abnormalities of keratin, hemidesmosomes, anchoring fibrils, anchoring filaments, and physicochemically-altered skin collagenase.^4^

As many as 23 EB variants are recognized and delineated based on their clinical appearance, extra coetaneous involvement, mode of inheritance, and level of tissue cleavage. These subtypes are in turn classified into 3 main groups based on the level of tissue separation that develops after mechanical trauma to the skin. Blistering occurs within the epidermis, within the basement membrane, or beneath the basement membrane in simplex, junctional, and dystrophic forms of inherited EB, respectively.^5^ Systemic features include bulla formation on hands, feet, elbows, and knees. The lesions are initially noted at or soon after birth. Bulla leave painful ulcers and, rupturing and healing are often followed by scarring and tissue contraction. Contractures and syndactyly of digits might result in the formation of claw-like hands. The upper esophagus frequently becomes stenotic, leading to dysphagia or esophageal obstruction.^6^

Oral features include repeated blistering and scar formation, leading to limited oral opening, ankyloglossia, and elimination of buccal and vestibular sulci, perioral structure, severe periodontal disease and alveolar bone resorption, atrophy of the maxilla with mandibular prognathism, an increased mandibular angle, and predisposition to oral carcinoma. Routine dental care or even normal tooth brushing might cause bulla on the oral mucosa.^7^ Painful blisters, restricted oral opening and poor manual dexterity as a result of finger deformities are factors contributing to diminished oral hygiene and delayed dental maturity.^8^ Satisfactory dental management of patients with EB represents one of the most difficult clinical challenges for the dentist as oral condition of these patients results in poor residual dentition and may lead to partial or complete edentulism.

Prosthetic rehabilitation of microstomia patients presents difficulties at all stages, from the preliminary impressions to fabrication of prostheses.^9^ Several techniques in patients with microstomia based on flexible, modified standard trays, sectioned trays,^10-11^ sectional ,^12^ and collapsible dentures have been proposed.^12-13^ In this clinical report, another simple method is presented, describing all stages from preliminary impressions to fabrication of complete dentures in single unit lined with laboratory soft liner, for a patient with limited mouth opening resulting from EB.

## Case Report

### Examination

**Figure 1:**
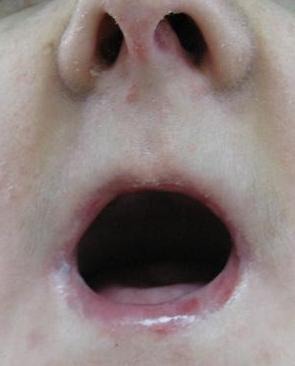
Microstomia of the patient.

A 21-year-old white woman, suffering from microstomia and poor manual ability resulting from epidermolysis bullosa was referred to the Department of Prosthodontics, Faculty of Dentistry, Tabriz University of Medical Sciences, for prosthetic rehabilitation. The diameter and circumference of her mouth were approximately 35 mm and 27 mm, respectively. The patient was diagnosed with recessive dystrophic EB (Figure 1), with no congenital missing of permanent teeth. The patient, however, had lost all teeth due to dental caries, as the limited oral opening had made it difficult to carry out restorative dental procedures.

**Figure 2:**
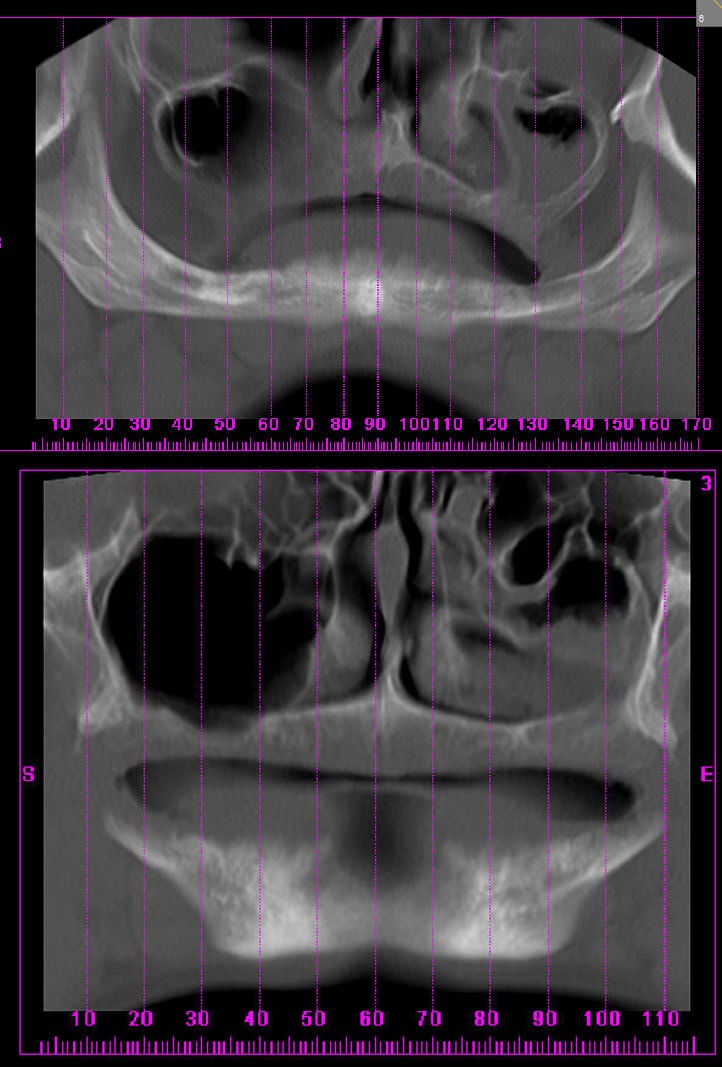
Computerized tomography view.

Two treatment options were considered: complete dentures and implant supported overdentures; treatment with dental implants was excluded because of volumetric deficiencies of bone in the CT scan analysis (Figure 2). Therefore, the selected treatment was conventional complete denture for upper and lower jaws.

### Prosthetic treatment procedure

Because of mechanical fragility of the skin with development of blisters and vesicles, resulting from minor mechanical friction or trauma,^14^ lubricating the patient’s lips and any other tissues susceptible to contact could help reduce the risk of shear forces and resulting tissue damage. Abundant and repeated lubrication of the lips with petroleum jelly is advised when performing any dental treatment on such patients.^15^

The use of a conventional impression tray, even the smallest one for primary impression, was impossible because of severe microstomia. Therefore, the heavy body putty silicon (Speedex, Coltene AG, Alstetten, Switzerland) was used for impression making of the residual ridge without any tray, but by two index fingers. The primary impression was removed after setting and observed for any voids and deficient areas (Figure 3a).


Figure 3. Preliminary impression from upper and lower jaws (a). Denture teeth custom-made from heat cured tooth-colored acrylic resin (b). Processed dentures (c). Relined dentures with silicone soft liner (d).

**a:**
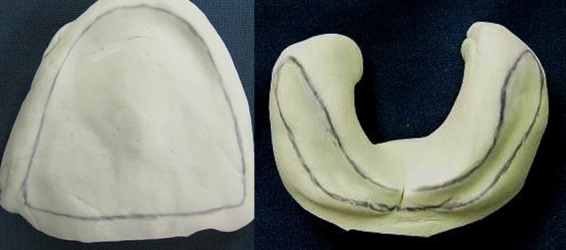

**b:**
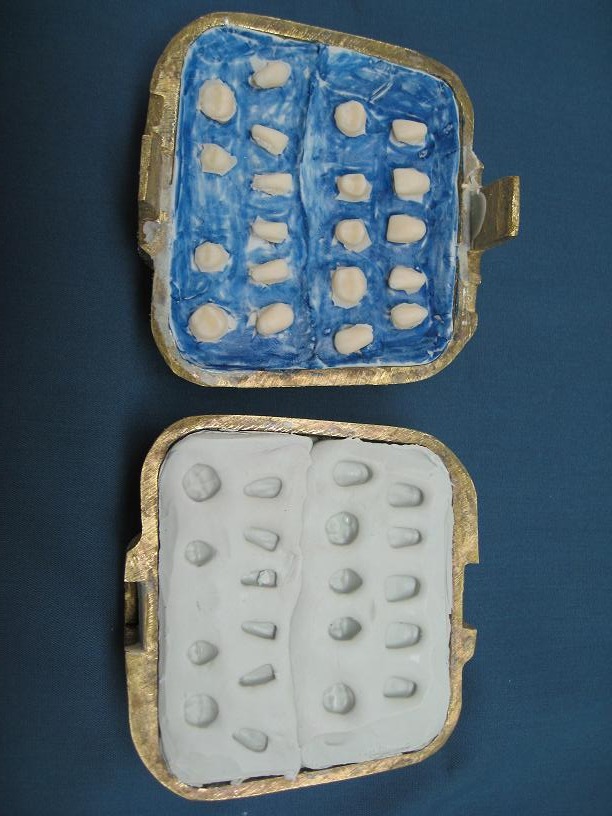

**c:**
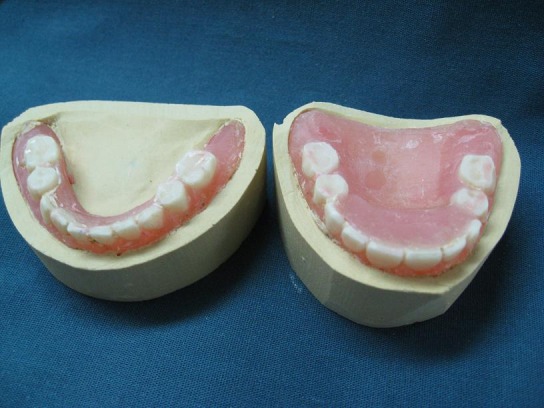

**d:**
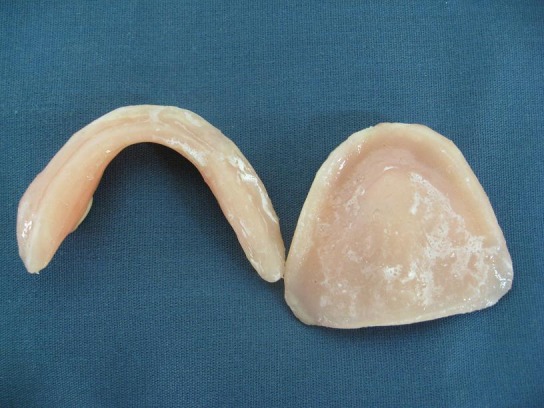

Preliminary impressions were poured and then custom trays were prepared with wax spacer for another more accurate maxillary and mandibular impression with fast-setting irreversible hydrocolloid (Tropicalgin, Zhermack, Italy). Custom trays were then prepared with wax spacer, with the flange of the custom trays adjusted 1 mm short of reflection areas. Because of the difficulty of sectional border molding due to limited mouth opening and fragile soft tissues, medium body polyether impression material (Impergum Soft, 3 Mespe AG, Seefeld, Germany) was used for final impression and border molding at the same time.

**Figure 4:**
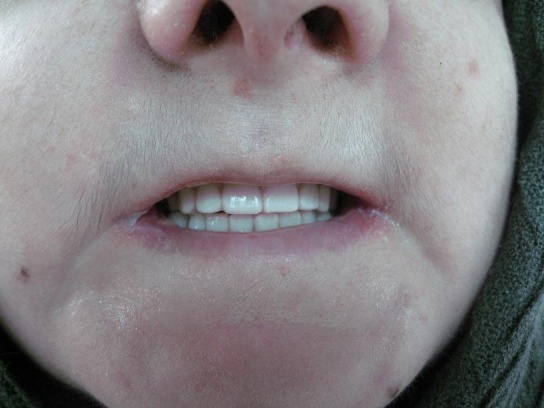
Final prosthesis in place.

The remaining procedures for preparation of complete dentures consisted of:

- Preparing occlusion rim and record base.
- Making a centric relation record and transferring the record from the patient to a nonadjustable articulator (monoplane concept).^16^
- Arrangement of the teeth. Because of a lack of denture teeth in appropriate sizes and shapes, conventional denture teeth were reshaped to appropriate sizes, duplicated and processed with heat-cured acrylic resin (Triplex Hot, Ivoclar Vivadent AG, FL Schochan/ Liechtenstein, Shade 27) (Figure 3b).
- Custom anterior teeth arrangement and obtaining patient approval.
- Custom prosthetic teeth arrangement and occlusion. Because of severe resorption in residual ridges, occlusal areas of posterior teeth were formed with 0 degree cusps.
- Denture processing and remounting (Figure 3c). Rigid denture base on fragile soft tissue was a challenge in this case; therefore, a long-term silicon soft liner (Permaflex Denture Reliner, Kohler, Neuhausen, Germany) was processed in soft tissue side of denture bases to reduce trauma (Figure 3d).
- Denture insertion in the oral cavity and observation for balanced occlusion (Figure 4).
- Oral and denture hygiene explanation. Oral care was constant irrigation with pure water or alkaline mouthrinses. Using a soft brush in conjunction with a very mild detergent or nonabrasive dentifrice was advised for cleansing the side of the dentures with soft liner.

### Follow-up sessions

The patient was visited 24 hours after the delivery of the dentures and the problems were corrected. The patient was followed again 72 hours, 1 week and 3 week later.

The patient was satisfied with the dentures and did not report any major difficulty in their application. The patient, however, continued a soft diet due to dysphagia.

## Discussion

Maintaining the dentition in patients with EB not only reduces the risk of soft tissue trauma to the mucosa and possibly to the esophagus through more efficient mastication without the formation of esophageal ulcers, but also results in improved nutrition, although satisfactory dental management of patients with EB represents one of the most difficult clinical challenges for the dentist.^17^ However, when maintenance of dentition is impossible, the use of endosseous implants in patients with edentulism might provide a considerably better outcome than traditional prosthetic methods.^17^ Fixed prostheses over implants can improve health, especially for totally edentulous patients, by allowing mastication of the alimentary bolus. In addition, patients do not need to remove and replace the prosthesis, avoiding the minor trauma to the mucosa that it may cause.^18^

It is believed that endosseous implants can be successfully placed and can provide support for removable prosthetic restorations in patients with epidermolysis bullosa. In fact, such treatment was found to improve patients’ quality of life considerably.^17^

In the present case, the use of implant-supported prosthesis was impossible because of economic considerations and ridge inadequacy. Therefore, construction of conventional complete denture was selected as the final treatment plan. In patients with microstomia due to EB, needing complete dentures, it is important that they have the ability of insertion and removal of their dentures. In this case, it was possible to render single-piece complete dentures treatment that has an advantage over complex prostheses in that the insertion and removal of complex denture prosthesis is an inconvenience for these patients with hands deformity. Although a lot of patients with microstomia may need two or more pieces in their prostheses or collapsible overdentures.^19^
